# Transcriptomic profile of cystic fibrosis airway epithelial cells undergoing repair

**DOI:** 10.1038/s41597-019-0256-6

**Published:** 2019-10-29

**Authors:** Alice Zoso, Aderonke Sofoluwe, Marc Bacchetta, Marc Chanson

**Affiliations:** Departments of Pediatrics, Gynecology & Obstetrics and of Cell Physiology & Metabolism, Geneva University Hospitals and Medical School of the University of Geneva, Geneva, Switzerland

**Keywords:** Apicobasal polarity, Respiratory distress syndrome, Transcriptomics

## Abstract

Pathological remodeling of the airway epithelium is commonly observed in Cystic Fibrosis (CF). The different cell types that constitute the airway epithelium are regenerated upon injury to restore integrity and maintenance of the epithelium barrier function. The molecular signature of tissue repair in CF airway epithelial cells has, however, not well been investigated in primary cultures. We therefore collected RNA-seq data from well-differentiated primary cultures of bronchial human airway epithelial cells (HAECs) of CF (F508del/F508del) and non-CF (NCF) origins before and after mechanical wounding, exposed or not to flagellin. We identified the expression changes with time of repair of genes, the products of which are markers of the different cell types that constitute the airway epithelium (basal, suprabasal, intermediate, secretory, goblet and ciliated cells as well as ionocytes). Researchers in the CF field may benefit from this transcriptomic profile, which covers the initial steps of wound repair and revealed differences in this process between CF and NCF cultures.

## Abstract (summary)

**Table:** 

Measurement(s)	messenger RNA
Technology Type(s)	RNA sequencing
Factor Type(s)	exposure to flagellin • cystic fibrosis versus non-cystic fibrosis • mechanical wounding
Sample Characteristic - Organism	Homo sapiens

Machine-accessible metadata file describing the reported data: 10.6084/m9.figshare.9944675

## Background & Summary

In this study, we compared by next generation RNA-sequencing (RNA-seq) the transcriptomic profile of human airway epithelial cells from cystic fibrosis (CF) patients and healthy donors (NCF). F508del, the most common variant of the CF transmembrane conductance regulator (*CFTR*) gene, is associated with a severe clinical phenotype that leads to chronic inflammation and infection of the airways by opportunistic pathogens, including *Pseudomonas aeruginosa*^1^. The continuous exposure to severe harmful stimuli places lungs at constant risk of injury and thereby, tissue repair is crucial for maintaining lung homeostasis^2,3^. CFTR plays a key role in regeneration of the airway epithelium, the repair of which is obviously insufficient to maintain lung functions in CF^4–10^. Knowledge of the molecular mechanisms regulating airway epithelial cell differentiation was mostly gained from lineage tracing studies in mouse models^3^. Less is known in human although application of single-cell RNA-seq on airway biopsies and primary HAEC cultures are rapidly filling up this gap^11–13^. The present work aims to identify gene expression changes in CF and NCF human airway epithelial cells (HAECs) undergoing repair. Some cultures of NCF and CF HAECs were also exposed to flagellin for 24 h to mimic *Pseudomonas aeruginosa* infection and processed for RNA-seq.

**Fig. 1:**
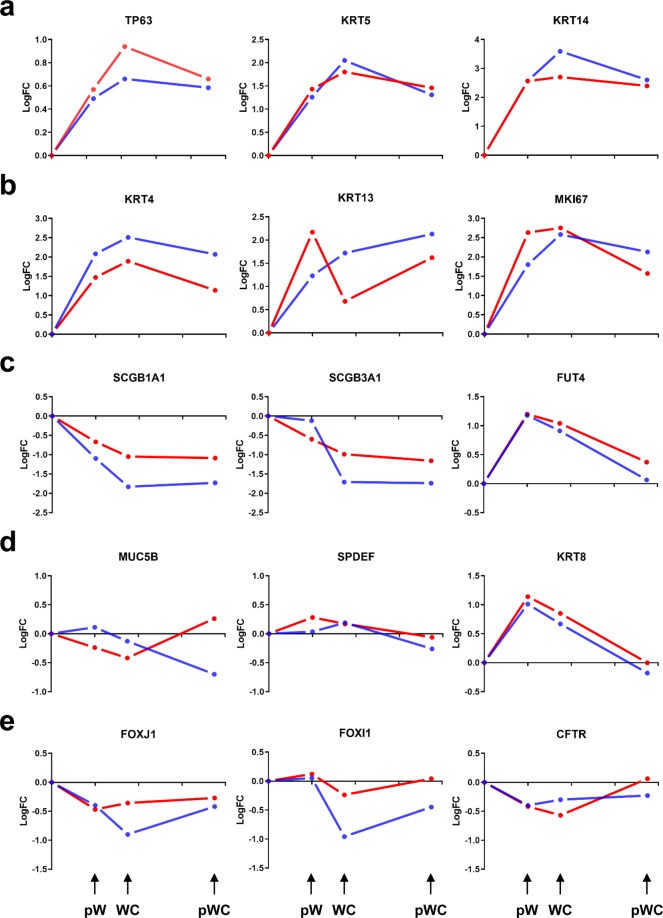
Changes in gene expression (logFC) of markers of subpopulations of NCF (blue lines and dots) and CF (red lines and dots) HAECs at different times of wound repair as compared to their initial expression (values set at 0) in non-wounded conditions. (**a**) Expression levels of basal cell marker genes: TP63, KRT5 and KRT14. (**b**) Expression levels of suprabasal cell marker genes (KRT4 and KRT13) and of a marker of cell proliferation (MKI67). (**c**) Expression levels of Club cell marker genes (SCGB1A1 and SCGB3A1), including the marker of immature cells (FUT4). (**d**) Expression levels of goblet cell marker genes (MUC5B and SPDEF) and of KRT8, which is a marker of early cell differentiation. (**e**) Expression levels of ciliated cell and ionocyte marker genes (FOXJ1 and FOXI1, respectively), with both subpopulations expressing CFTR. Data are expressed as means; error bars were not drawn for clarity since no statistical differences were observed between NCF and CF cultures. pW: post wounding; WC: wound closure; pWC; post wound closure.

**Fig. 2:**
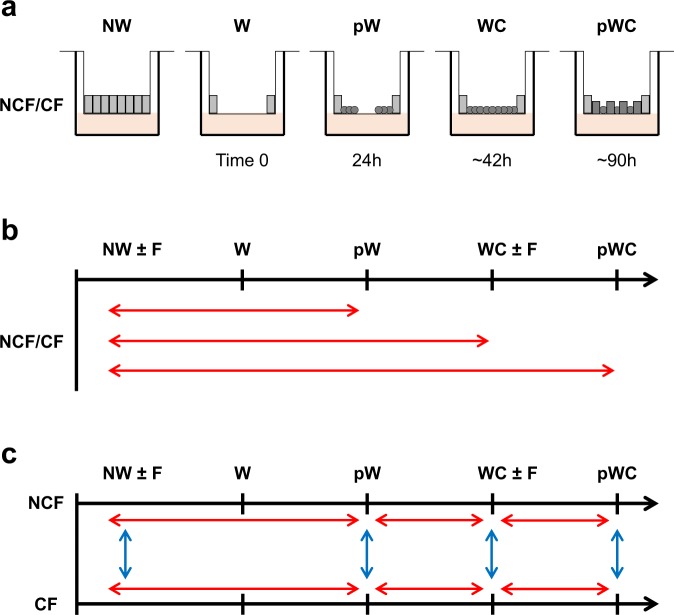
Experimental design and condition’s comparison. (**a**) Schematic illustration of the wound-induced repair process in HAECs. Well-differentiated airway epithelium 3D cultures from CF patients and NCF donors were used, corresponding the non-wounded (NW) condition. At time 0, a circular wound (W) was induced in the center of the culture but leaving intact the epithelium at the periphery. Twenty-four hours after wounding (pW), migrating and proliferating cells started to cover the denuded area. Wound closure (WC) was reached 42 hours after wounding. mRNA was isolated from two Transwells per patient/donor and for each condition, NW, pW, WC and 48 hours after wound closure (pWC; 90 hours after wounding). In parallel experiments, 2 NW and WC Transwells per patient/donor were treated with flagellin to mimic infection with *Pseudomonas aeruginosa*. (**b**) Illustration of the gene expression comparisons performed between different conditions after wounding (pW, WC, pWC) and the initial NW condition, exposed or not to flagellin (F). (**c**) Illustration of the gene expression comparisons performed for all conditions between CF and NCF cultures.

The tracheobronchial airway epithelium is pseudostratified and constituted of basal (BCs), secretory Club/Clara (SCs), ionocytes (ICs), mucin-producing goblet (GCs) and ciliated cells (CCs)^3,11,12,14^. It is well demonstrated that epithelium regeneration/repair is initiated by BC proliferation to repopulate the denuded injured area^3^. In parallel, subsets of progenitor cells (suprabasal cells, sBCs) cycle and/or progressively mature to intermediate - or early progenitor - cells leading to the generation of SCs. After wound closure, all cells exit the cell cycle, BCs return to their original state while SCs terminate their differentiation to GCs and CCs. Figure 1 illustrates the logFC changes in expression of markers of the different cell subtypes with time of repair after injury of CF and NCF HAEC primary cultures. We focused on the initial steps of repair by comparing the post-wounding conditions (24 h post-wounding pW, wound closure WC, usually reached 42 hours after injury, and 2-days post wound closure pWC) to the control non-wounded condition (NW). We monitored TP63, cytokeratin 5 (KRT5) and KRT14 for BCs (Fig. 1a), KRT4 and KRT13 for sBCs (Fig. 1b), SCGB1A1 and SCGB3A1 for SCs (Fig. 1c), MUC5B and SPDEF for GCs (Fig. 1d), FOXJ1, FOXI1 and CFTR for CCs and ICs (Fig. 1e). Globally, proliferation can be evaluated by the expression of MKI67 (Fig. 1b) and early differentiation by the expression of KRT8 (Fig. 1d), a marker which is not detected in BCs and sBCs. Note that FUT4, a marker of immature SCs is detected (Fig. 1c). The results indicate that the repair process is engaged after wounding in both CF and NCF cultures and that our RNA-seq allows monitoring gene expression during the initial steps before the generation of mature SCs. A schematic overview of the experimental conditions as well as the comparisons performed between conditions and groups are provided in Fig. 2. Table 1 indicates the number of gene changes for each time point after wounding relative to the NW conditions (top). Comparison of the number of gene changes between conditions (pW vs NW; WC vs pW; pWC vs WC) is also given (middle). We also performed comparison of gene changes between CF and NCF HAEC cultures for the different conditions (bottom). Again, up- and downregulated genes in CF HAECs are detected for all conditions, suggesting alterations in the switch between proliferation and differentiation for CF HAECs. Finally, flagellin stimulation at Time 0 (NW) and at WC further highlighted differences in the transcriptomic response of CF HAECs (Table 2).

**Table 1:** Number of differently expressed genes with FDR (False Discovery Rate) 5% and the number of which have a fold-change 2 (FC 2) thresholds.

	# up-regulated genes	# down-regulated genes	No change	# with FC 2	of which # FC < 2	of which # FC > 2
**Compare different Times per Group**						
NCF						
pW vs NW	2930	2359	9669	1260	339	921
WC vs NW	3871	3459	7628	2148	791	1357
pWC vs NW	630	127	14201	305	15	290
CF						
pW vs NW	3297	3142	8519	1244	360	884
WC vs NW	3109	2881	8968	1136	266	870
pWC vs NW	474	58	14426	195	3	192
**Compare different Conditions per group**						
NCF						
pW vs NW	2930	2359	9669	1260	339	921
WC vs pW	57	15	14886	32	2	30
pWC vs WC	517	1414	13027	410	358	52
CF						
pW vs NW	3297	3142	8519	1244	360	884
WC vs pW	0	0	14958	0	0	0
pWC vs WC	212	668	14078	141	107	34
**Compare different Groups per Condition**						
CF vs NCF						
NW	181	110	14667	162	40	122
pW	47	86	14825	95	73	22
WC	217	480	14261	309	241	68
pWC	55	114	14789	111	80	31

(Top) Comparisons between different times of HAEC repair with initial, non-wounded condition, for NCF and CF cultures. (Middle) Comparisons between different times of HAEC repair for NCF and CF cultures. (Bottom) Comparisons between NCF and CF cultures for the different times of HAEC repair. NW, non-wounded; pW, 24 h post-wound; WC, wound closure; pWC, 2d post-wound closure.

**Table 2:** Number of differently expressed genes with FDR (False Discovery Rate) 5% and the number of which have a fold-change 2 (FC 2) thresholds.

	# up-regulated genes	# down-regulated genes	No change	# with FC 2	of which # FC < 2	of which # FC > 2
**Compare different Times per Group**						
NCF + F						
WC vs NW	1292	624	14082	656	122	534
CF + F						
WC vs NW	1302	668	14028	647	142	505
**Compare different Conditions per group**						
NCF ± F						
NW	643	70	15285	496	35	461
WC	31	14	15953	37	13	24
CF ± F						
NW	564	189	15245	438	83	355
WC	222	34	15742	183	21	162
**Compare different Groups per Condition**						
CF + F vs NCF + F						
NW	14	140	15844	144	135	9
WC	9	38	15951	43	38	5

(Top) Comparisons between wound closure (WC) of HAEC repair and the initial, non-wounded (NW) condition, for NCF and CF cultures treated with flagellin (F). (Middle) Comparisons between flagellin (F)-treated and non-treated NCF and CF cultures that were not wounded (NW) and at time of wound closure (WC) of HAEC repair. (Bottom) Comparisons between NCF and CF non-wounded (NW) cultures and time of wound closure (WC) of HAEC repair after flagellin (F) exposure.

In summary, this study presents RNA-seq data from healthy and CF human HAECs undergoing repair after injury. We extracted gene expression of typical marker genes of the different cell subtypes that constitute the airway epithelium and report differences in the repair process between CF and NCF cultures. We believe that these data will be valuable for researchers studying airway epithelium regeneration in the context of the CF disease.

## Methods

### Cell cultures

Well-differentiated primary cultures of bronchial airway epithelial cells (MucilAir™ and MucilAir™-CF) on Transwell filters at the air-liquid interface for 45–60 days were purchased from Epithelix Sàrl (Plan-les-Ouates, Switzerland). All CF HAEC cultures were generated from 7 patients homozygous for the F508del CFTR variant. NCF cultures were generated from 7 subjects but one culture (subject 4) did not differentiate appropriately and was discarded. Detailed characteristics of the patients (age, sex, smoking status) are not available. The basal medium, which consisted of DMEM:F12 (3:1, Life Technologies, Zug, Switzerland) supplemented with 1.5% Ultroser G (Bioserpa, Cergy, France) and antibiotics, was refreshed every 2 days. Mechanical wounding was performed using an airbrush linked to a pressure regulator, as previously described^15^.

### RNA extraction

Total RNA was extracted using Qiagen RNeasy Kit (Qiagen, Hombrechtikon, Switzerland), according to the manufacturer’s instructions. At 24 hours post-wound (pW) and at WC, the Transwell filters were cut off and undamaged cells at the periphery of the wound were discarded from the repairing cells using a sterile scalpel before lysis and RNA extraction. Two filters were pooled per condition. RNA-seq was performed by the *i*GE3 Genomic Platform at the Faculty of medicine, University of Geneva.

### Differential gene expression analysis

Library size normalizations and differential gene expression calculations have been performed using the package edgeR (http://www.ncbi.nlm.nih.gov/pmc/articles/PMC2796818/). The genes having a count above one count per million reads (cpm) in at least 3 samples were kept for the analysis. For each comparison, the latest condition was used as the ‘control’, i.e. genes with a positive fold-change are more expressed in the first condition compared to the ‘control’ condition. Genes with maximal expression value in any of the compared conditions lower than 1 RPKM (reads per kb per million read) were removed from the analysis before calling for differentially expressed genes. The differentially expressed gene tests were done with a general linear model with a negative binomial distribution. The differentially expressed genes p-values are corrected for multiple testing error with a 5% FDR (false discovery rate) and the correction used is Benjamini-Hochberg (BH). By default, the fold-change (FC) and the Benjamini-Hochberg corrected p-value thresholds were set to 2 and 0.01, respectively. Genes with higher Benjamini-Hochberg corrected p-value or lower FC were not considered as differentially expressed.

## Data Records

The data can be accessed to NCBI Gene Expression Omnibus (GEO) with the accession number GSE127696^16^. The lists of differentially expressed genes with FDR 5% and FC 2 thresholds for the comparisons indicated in Tables 1 and 2 are available in figshare^17^. Datasets of original reads for all conditions (NCF and CF, before and after wounding) are available in the NCBI SRA repository^18^.

## Technical Validation

### RNA integrity assessment

Before sequencing, QuBit (Invitrogen) was used to assess RNA quality and quantity without prior purification of the samples.

### RNA-seq data quality assessment

**Fig. 3:**
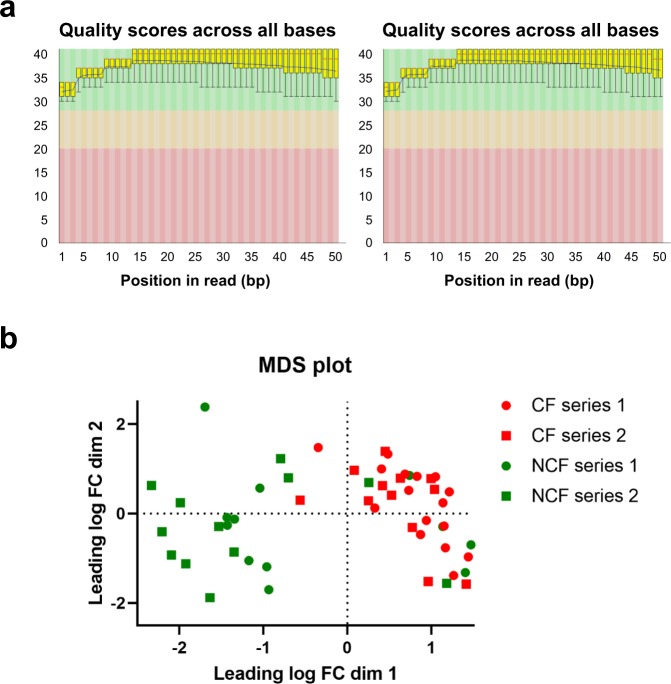
Quality assessment FASTQ data. (**a**) The quality distribution along the reads plot is shown for one NCF (left, sample 54) and one CF (right, sample 26) sample. Box and whisker plots demonstrate the distribution of per base quality for each left and right read position read for each of the analyzed samples. Mean value is indicated by the dark line; the yellow box represents the interquartile range (25–75%) with the lower and upper whiskers representing the 10 and 90% points, respectively. (**b**) MDS (principal components analysis) plot indicating the similarity of the counts in the samples obtained from the first (black letters) and the second (red letters) series of NCF and CF cultures.

Single read of 50 bases, TruSeq stranded mRNA, was performed with a HiSeq 4000 from Illumina. The sequencing quality control was done with FastQC (http://www.bioinformatics.babraham.ac.uk/projects/fastqc/). Quality scores of 32–40 were achieved (Fig. 3a), corresponding to 1/1000 and 1/10’000 chance of errors, respectively. The reads were mapped with STAR, an ultra-fast and universal RNA-seq aligner, which can do spliced alignments and read clipping: http://bioinformatics.oxfordjournals.org/content/early/2012/10/25/bioinformatics.bts635.

Only the reads that are mapped once to the genome are considered for the read allocation to genomic features. Ambiguous reads were removed using featureCounts: http://www.ncbi.nlm.nih.gov/pubmed/24227677.

Reads mapping is provided as a Supplementary Data File (Online-only Table 1). For polyA-enriched RNAseq, 70% or more reads uniquely assigned to a gene are considered really good, although this percentage may be affected by the nature of the different expressed genes.

Sequencing was performed on two different occasions with RNA samples collected at one-year interval times. Figure 3b shows the multi-dimensional scaling (MDS) plot (principal components analysis) of the samples, which gives an indication of the similarity of the counts in the earlier and former experiments (first and second series, respectively). No batch effect could be observed between the two sequenced data.

## Online-only Table

**Online-only Table 1:** Mapping with STAR and reads allocation to genomic features.

Conditions	Mapping status				Allocation status			
	Initial	Unique	Multiple	Unmapped	Ambiguity	noFeatures	Assigned	Assigned (%)
Sample 1: CF_pW_rep1	27055856	22084611	4572500	398745	417070	1701042	19966499	73.8
Sample 2: CF_pW_rep2	29375070	25107286	3922319	345465	497780	2343725	22265781	75.8
Sample 3: CF_pW_rep3	25831778	21983510	3607914	240354	412046	2083512	19487952	75.44
Sample 4: CF_pW_rep4	26472753	22580726	3577899	314128	413227	2005814	20161685	76.16
Sample 5: CF_pW_rep5	24530221	21110696	3361651	57874	396777	2529895	18184024	74.13
Sample 6: CF_pW_rep6	18263626	16046779	2155259	61588	313639	1671889	14061251	76.99
Sample 7: CF_pW_rep7	23098321	20140508	2891639	66174	414076	1766582	17959850	77.75
Sample 8: CF_pWC_rep1	28343765	23391672	4634526	317567	418971	2195260	20777441	73.31
Sample 9: CF_pWC_rep2	28421910	23499099	4460243	462568	420870	2665892	20412337	71.82
Sample 10: CF_pWC_rep3	32049392	27330970	4437995	280427	482346	3058976	23789648	74.23
Sample 11: CF_pWC_rep4	29426653	24568958	4527872	329823	437617	2350594	21780747	74.02
Sample 12: CF_pWC_rep5	23509435	20325015	3118066	66354	363314	3093031	16868670	71.75
Sample 13: CF_pWC_rep6	26808287	23222520	3499746	86021	417070	3306680	19498770	72.73
Sample 14: CF_pWC_rep7	25390525	21972218	3337373	80934	406602	3151580	18414036	72.52
Sample 15: CF_Flag_NW_rep1	28937956	23995951	4593671	348334	457062	2263768	21275121	73.52
Sample 16: CF_Flag_NW_rep2	27896159	23081832	4484454	329873	429904	2639009	20012919	71.74
Sample 17: CF_Flag_NW_rep3	29012044	22853960	5806857	351227	430473	2819945	19603542	67.57
Sample 18: CF_Flag_NW_rep4	29966891	25029508	4578373	359010	454832	2781231	21793445	72.73
Sample 19: CF_Flag_NW_rep5	25243568	21799921	3370111	73536	399795	3530522	17869604	70.79
Sample 20: CF_Flag_NW_rep6	23525961	20540760	2906760	78441	375115	3032076	17133569	72.83
Sample 21: CF_Flag_NW_rep7	25429057	22171330	3164973	92754	405822	3454333	18311175	72.01
Sample 22: CF_Flag_WC_rep1	27455756	22812623	4276541	366592	422674	1936924	20453025	74.49
Sample 23: CF_Flag_WC_rep2	26052335	22610952	3219507	221876	407641	2206644	19996667	76.76
Sample 24: CF_Flag_WC_rep3	29676536	24970137	4282099	424300	488602	1975516	22506019	75.84
Sample 25: CF_Flag_WC_rep4	27028337	22848427	3820941	358969	421962	1998139	20428326	75.58
Sample 26: CF_Flag_WC_rep5	26357220	22209036	4079640	68544	414080	2735889	19059067	72.31
Sample 27: CF_Flag_WC_rep6	20339155	17902816	2367795	68544	334978	2371446	15196392	74.71
Sample 28: CF_Flag_WC_rep7	20846068	17868257	2908468	69343	332474	2297950	15237833	73.1
Sample 29: CF_NW_rep1	27354203	22701945	4355935	296323	393776	1914877	20393292	74.55
Sample 30: CF_NW_rep2	28989623	24338336	4366492	284795	421404	2968003	20948929	72.26
Sample 31: CF_NW_rep3	25604382	21534527	3759304	310551	377236	2375862	18781429	73.35
Sample 32: CF_NW_rep4	28117785	23502033	4217864	397888	418353	2317895	20765785	73.85
Sample 33: CF_NW_rep5	25800266	22277199	3446004	77063	401969	3890419	17984811	69.71
Sample 34: CF_NW_rep6	24229957	21197246	2966851	65860	385978	2942315	17868953	73.75
Sample 35: CF_NW_rep7	19389289	16853083	2469615	66591	292314	2848474	13712295	70.72
Sample 36: CF_WC_rep1	27713621	22242514	5101546	369561	415331	1750702	20076481	72.44
Sample 37: CF_WC_rep2	27795476	23032050	4477032	286394	430414	2328828	20272808	72.94
Sample 38: CF_WC_rep3	27297648	23085529	3808956	403163	447701	1887015	20750813	76.02
Sample 39: CF_WC_rep4	27518854	23301353	3882416	335085	430413	1883012	20987928	76.27
Sample 40: CF_WC_rep5	20285541	17495579	2736494	53468	340579	2117350	15037650	74.13
Sample 41: CF_WC_rep6	16307572	14199530	2065283	42759	265512	1601438	12332580	75.62
Sample 42: CF_WC_rep7	20996505	18300489	2621865	74151	328438	2462407	15509644	73.87
Sample 43: NCF_pW_rep1	27294957	22843894	4143637	307426	437924	2091351	20314619	74.43
Samples 44: NCF_pW_rep2	28368473	23984765	4154289	229419	468951	2113842	21401972	75.44
Samples 45: NCF_pW_rep3	29954171	25078264	4583543	292364	475086	2602827	22000351	73.45
Samples 46: NCF_pW_rep5	20513223	17764819	2695539	52865	338937	2047538	15378344	74.97
Samples 47: NCF_pW_rep6	23061659	19990115	3013511	58033	390696	2407910	17191509	74.55
Samples 48: NCF_pW_rep7	20620005	17871570	2691218	57217	347867	1792089	15731614	76.29
Samples 49: NCF_pWC_rep1	26505296	22252175	3985854	267267	399269	2095608	19757298	74.54
Samples 50: NCF_pWC_rep2	29119661	24286641	4548069	284951	452589	2590125	21243927	72.95
Samples 51: NCF_pWC_rep3	23987320	19882776	3867828	236716	369674	1914042	17599060	73.37
Samples 52: NCF_pWC_rep5	22980476	19244779	3674432	61265	342536	2601439	16300804	70.93
Samples 53: NCF_pWC_rep6	26682850	22982851	3616350	83649	430766	3802346	18749739	70.27
Samples 54: NCF_pWC_rep7	20019647	16557495	3408641	53511	309569	2251749	13996177	69.91
Samples 55: NCF_FlagNW_rep1	31537158	27156134	3722713	658311	496149	2360464	24299521	77.05
Samples 56: NCF_FlagNW_rep2	25992915	20158038	5576651	258226	353978	2099575	17704485	68.11
Samples 57: NCF_FlagNW_rep3	26241469	22052164	3881570	307735	424212	2330329	19297623	73.54
Samples 58: NCF_FlagNW_rep5	23661828	20567572	3018174	76082	372910	3604790	16589872	70.11
Samples 59: NCF_FlagNW_rep6	25975904	22637672	3268895	69337	429978	2889146	19318548	74.37
Samples 60: NCF_FlagNW_rep7	24019200	20562887	3394438	61875	387713	2669143	17506031	72.88
Samples 61: NCF_FlagWC_rep1	27322955	21568566	5486648	267741	381699	1992246	19194621	70.25
Samples 62: NCF_FlagWC_rep2	28392971	23251231	4930663	211077	433337	2366930	20450964	72.03
Samples 63: NCF_FlagWC_rep3	30173946	26023032	3829860	321054	489253	2749847	22783932	75.51
Samples 64: NCF_FlagWC_rep5	25019094	21458446	3494641	66007	397230	2788270	18272946	73.04
Samples 65: NCF_FlagWC_rep6	20993388	18066453	2871509	55426	352920	1881789	15831744	75.41
Samples 66: NCF_FlagWC_rep7	25825712	21766171	3991489	68052	407328	2489378	18869465	73.06
Samples 67: NCF_NW_rep1	26440644	22292672	3883826	264146	412080	2278593	19601999	74.14
Samples 68: NCF_NW_rep2	28230517	24150322	3837544	242651	433291	2790739	20926292	74.13
Samples 69: NCF_NW_rep3	28221303	23337680	4653126	230497	422161	2669611	20245908	71.74
Samples 70: NCF_NW_rep5	25201987	21326171	3807662	68154	392759	3046604	17886808	70.97
Samples 71: NCF_NW_rep6	21834253	18736227	3036005	62021	361539	2330198	16044490	73.48
Samples 72: NCF_NW_rep7	25440769	22193920	3175802	71047	410317	3077105	18706498	73.53
Samples 73. NCF_WC_rep1	26289846	21571767	4417932	300147	394181	1666345	19511241	74.22
Samples 74: NCF_WC_rep2	26811590	22682744	3895770	233076	411541	2375317	19895886	74.21
Samples 75: NCF_WC_rep3	26602370	22751639	3594974	255757	413537	2351103	19986999	75.13
Samples 76: NCF_WC_rep5	18082081	15522930	2510520	48631	280435	1694281	13548214	74.93
Samples 77: NCF_WC_rep6	27839732	23992391	3773367	73974	452694	2742183	20797514	74.7
Samples 78: NCF_WC_rep7	17914419	15257942	2611919	44558	287221	1494456	13476265	75.23
